# Can the level of copper in the hippocampus witness type-II diabetes versus Alzheimer's disease?

**DOI:** 10.1016/j.ebiom.2022.104403

**Published:** 2022-12-13

**Authors:** Christelle Hureau

**Affiliations:** LCC-CNRS, Université de Toulouse, CNRS, Toulouse, France

**Keywords:** sAD, sporadic Alzheimer's Disease, Aβ, amyloid-β

Copper is an essential trace metal ion involved in many key physiological processes and is the co-factor of the related enzymes, including the cytochrome c oxidase of the respiratory chain and the superoxide dismutases that takes part in the fight against oxidative stress. As for most of other trace elements, a proper copper homeostasis is crucial for a good health.^1^ Copper deficiency or excess represents pathologic conditions, as exemplified by the two genetic disorders, Menkes and Wilson diseases corresponding to a failure in copper absorption and distribution and to a failure in copper excretion, respectively.^2^ Interestingly, both diseases induce several neurological consequences. This perfectly illustrates that copper has a “Janus-type” behavior: when correctly placed and bound to the right enzymes, copper is essential for the normal functioning of the human body but when misplaced and/or unbound by dedicated enzymes and proteins, copper becomes toxic.^3^ The toxicity is mainly related to the redox ability of the copper ions that can switch between the +I and +II states, thus participating in the production of reactive oxygen species, linked to oxidative stress.

Emerging evidences indicate a connection between altered copper levels and various neurodegenerative pathologies, mainly Alzheimer's disease (AD) but also Parkinson's disease and amyotrophic lateral sclerosis.^4^ In fact, in AD, an aberrant copper homeostasis is a widely accepted hypothesis, with however divergent reports about a copper excess or a copper deficiency. A current vision reconciles such apparent results proposing an increase in the labile pool of copper and a decrease in the protein-bound copper.^5^ Hence speciation of copper matters.

Type-2 Diabetes Mellitus (T2DM) is a metabolic disorder linked to overweight, life style and genetic factors and triggers several complications such as cardiovascular diseases. T2DM may also contribute to neurodegeneration via a variety of paths, including a possible cross-talk with AD leading to the currently so-called type-3 diabetes hypothesis.^6^ Last, copper has been linked to type-2 diabetes Mellitus (T2DM), with a reported increased level in the peripheral circulation of cerulosplasmin-unbound labile copper pool.^7^

In a recent issue of eBioMedicine, Cooper and coworkers evidenced an elevated level of total copper content in the hippocampus on T2DM brains,^8^ up to level reported for untreated Wilson Disease. The results of the study relies on a limited sample size (6 brains for T2DM and 6 controls), due to the intrinsic difficult accessibility to the biological material. The copper level increase is location specific (no increase were observed in the other brain areas probed). Perturbations in the levels of other essential inorganic ions, including zinc and iron, were not observed, thus pointing out to a metal specific effect. This seminal study thus proposes a possible role of copper in neurodegeneration associated to T2DM that could be linked, among other, to the copper redox ability and its participation to oxidative stress.

Using the same methodology, Cooper and coworkers detect a decrease in the average copper level in sAD hippocampus.^9^ If one considers that for both diseases similar elevation trends of copper level in cerulosplasmin unbound copper was reported, such a result was unexpected.^7^ Because the variation in the copper level with respect to control population were elevated in case of T2DM^8^ and decreased in AD,^9^ the copper-induced toxicity *must* proceed via distinct mechanisms, reminiscent of what is described for Wilson and Menkes diseases. Such mechanisms still have to be fully elucidated. However, in AD, beyond the copper level, its extra- versus intra-cellular localization is very important, with increases in the extracellular space, especially in the amyloid deposits where copper is bound to the Aβ peptides (up to mM concentration). Hence, the results found here of elevated hippocampus copper level in T2DM may also be compared to what is found in the extracellular medium in AD. Being able to differentiate between the various copper species and localization thus appears as a further requirement to shed light on the complicated role of copper in T2DM and AD.

The discovery of Cooper makes two main questions arise. T2DM is proposed as a risk factor of AD based on recent epidemiological data.^6^ It was proposed that the assembly of the amyloid-forming peptide Aβ regarded as an early and crucial event in the etiology of AD was enhanced in T2DM. Besides, copper modulates Aβ assembly towards more toxic species. Hence, beyond its acknowledged impact linked to oxidative stress-induced toxicity, copper might also connect the two diseases via the modulation of Aβ assembly by T2DM-induced copper-overload. Copper ions can be removed using dedicated molecules, named chelators or metallophores.^10^ This is a therapeutic option currently under focus in case of AD and Wilson Disease. Taken into consideration that the pool of unessential copper should be targeted (*id est* labile copper not bound to proteins and enzymes), such strategy might also be appropriate in case of T2DM induced copper hippocampus overload.

## Contributors

The authors contributed as follows: Literature search: C.H. and Writing: C.H.

## Declaration of interests

The authors declare no conflict of interest.
